# Coronavirus disease 2019 and lung cancer: where are we?

**DOI:** 10.37349/etat.2023.00182

**Published:** 2023-10-30

**Authors:** Abrahams Ocanto, Xabier Mielgo-Rubio, Javier Luna Tirado, Nuria Linares Mesa, Marta López Valcárcel, Sara Pedraza, Victoria Vera Barragan, Patricia Valencia Nieto, Juan Zafra Martín, Felipe Couñago

**Affiliations:** Istituto Nazionale Tumori-IRCCS-Fondazione G. Pascale, Italy; ^1^Department of Radiation Oncology, Hospital Universitario San Francisco de Asís, GenesiCare Madrid, 28002 Madrid, Spain; ^2^Department of Radiation Oncology, Hospital Universitario Vithas La Milagrosa, GenesiCare Madrid, 28002 Madrid, Spain; ^3^Department of Medical Oncology, Hospital Universitario Fundación Alcorcón, 28922 Madrid, Spain; ^4^Department of Radiation Oncology, Hospital Universitario Fundación Jiménez Díaz, 28040 Madrid, Spain; ^5^Department of Radiation Oncology, Hospital Universitario Juan Ramón Jiménez, 21005 Huelva, Spain; ^6^Department of Radiation Oncology, Hospital Universitario Puerta de Hierro, 28222 Madrid, Spain; ^7^Department of Radiation Oncology, Hospital Universitario 12 de Octubre Madrid, 28041 Madrid, Spain; ^8^Department of Radiation Oncology, Hospital Universitario de Badajoz, 06080 Badajoz, Spain; ^9^Department of Radiation Oncology, Hospital Clínico Universitario de Valladolid, 47003 Valladolid, Spain; ^10^Group of Translational Research in Cancer Immunotherapy, Centro de Investigaciones Médico-Sanitarias (CIMES), Universidad de Málaga (UMA), Instituto de Investigación Biomédica de Málaga (IBIMA), 29010 Málaga, Spain; ^11^Department of Radiation Oncology, Hospital Universitario Virgen de la Victoria, 29010 Málaga, Spain; ^12^Department of Radiation Oncology, Emilio Vargas, GenesisCare Madrid, 28002 Madrid, Spain

**Keywords:** Coronavirus disease 2019, lung cancer, radiotherapy

## Abstract

Oncology patients are more susceptible to severe acute respiratory syndrome coronavirus 2 (SARS-CoV-2) infection due to hospital contact and an immunological system that can be compromised by antineoplastic therapy and supportive treatments. Certain similarities have been described in the physiopathology of coronavirus disease 2019 (COVID-19) and lung cancer (LC) that may explain the higher probability of these patients of developing a more serious disease with more frequent hospitalizations and even death, especially with the addition of smoking, cardiovascular and respiratory comorbidities, old age and corticosteroids use. Pre-existing lesions and cancer therapies change the normal architecture of the lungs, so diagnostic scales such as COVID-19 Reporting and Data System (CO-RADS) are of vital importance for a correct diagnosis and patient homogenization, with a high inter-observer correlation. Moreover, anticancer treatments have required an adaptation to reduce the number of visits to the hospital [hypofractionated radiotherapy (RT), larger intervals between chemotherapy cycles, delay in follow-up tests, among others]. In a way, this has also caused a delay in the diagnosis of new cancers. On the other hand, vaccination has had a positive impact on the mortality of these patients, who maintain a similar seroprevalence to the rest of the population, with a similar impact in mortality.

## Introduction

Severe acute respiratory syndrome coronavirus 2 (SARS-CoV-2) is an RNA virus of the Coronaviridae family [1]. It is highly transmittable and responsible of at least 600 million cases and around 6 million deaths [2]. The infection by SARS-CoV-2 is denominated as coronavirus disease 2019 (COVID-19) a comprises a variable range of symptoms, from an asymptomatic state to mild respiratory symptoms and even severe pneumonia with acute respiratory distress syndrome (ARDS) [1].

Cancer patients are more susceptible to infections and lung cancer (LC) is no exception, as increased severity with high mortality has been reported in this subgroup of patients [3]. For this reason, it is important to know the characteristics of patients affected by COVID-19, the impact in disease diagnosis, specific radiological findings, frequent complications and the impact in the natural history of their cancer and the needed adaptation of antineoplastic treatments during the pandemic.

## Physiopathology of COVID-19 in LC patients

The incidence and severity of COVID-19 infection are higher in cancer patients. Particularly, LC patients are more vulnerable to the infection and register higher mortality, hospitalization and complication rates compared to other neoplasms [4–6].

SARS-CoV-2 is a single-stranded RNA virus that enters host cells through the union of the spike (S) protein with the receptor of the angiotensin-converting enzyme 2 (ACE2), which is mostly expressed in the lungs but is also present in other organs such as the heart, kidneys, intestine and brain. This process is regulated by the transmembrane protease serine 2 (TMPRSS2). Once inside the cell, SARS-CoV-2 loses its capsid and liberates its genome to the cytoplasm, initiating the replication of viral RNA. Generally, during this first phase the individual is either asymptomatic or suffers from cold-like symptoms. In most COVID-19 cases, the immune system is able to suppress viral replication and contain the infection. However, the disease may progress to a second phase characterized by an overactivation of the immune system. In this phase, some patients can develop an immune reaction against lung parenchyma, causing parenchymal and endothelial damage that can lead to the development of a pneumonia characterized by mainly peripherical ground-glass opacities as radiological findings and a state of severe hypoxemia clinically [7].

There are similarities between the physiopathology of COVID-19 and LC that seem to explain the increased risk of infection in these patients. There are reports of the association between ACE2 and TMPRSS2 with LC. Although results are not very conclusive, these seem to be overexpressed in smoker with LC, making them more susceptible to SARS-CoV-2 infection [8, 9]. Moreover, mutations in the genes that codify both proteins could be associated with carcinogenesis given their correlation with regulatory genes of epithelial-mesenchymal transition (EMT) [10]. Higher levels of plasminogen activator inhibitor-1 (PAI-1) have been observed in patients with severe COVID-19, which promotes a coagulopathy and secretion of proinflammatory cytokines. PAI-1 also promotes carcinogenesis in LC and has therefore been proposed as a new biomarker [8, 10].

The proinflammatory state, crucial in SARS-CoV-2 infection, is also characteristic of LC and respiratory diseases. It is widely known that the inflammation caused by tobacco or certain lung diseases is associated with LC. In this context, interleukin-6 (IL-6) modulates the tumor microenvironment and high levels have been associated with advanced disease and higher risk of brain metastases. Its role is also important in COVID-19, as high levels are correlated with severe symptoms and mortality [7, 11].

Reactive oxygen species (ROS) also have a paramount role in both diseases. The activation of hypoxia-inducible factor-1 (HIF-1) and nuclear factor kappa-B (NF-κB) pathways lead to an overactivation of the immune system that exacerbates inflammation in COVID-19. It has been suggested that the overexpression of HIF-1 promotes cancer development as well as metastatic dissemination due to increased angiogenesis in the context of hypoxia. Furthermore, NF-κB activation in T cells suppresses tumor growth [12]. Mechanisms that may or may not favor carcinogenesis in COVID-19 are summarized in Table 1.

**Table 1:** Mechanisms in favor and against COVID-induced carcinogenesis

**Against transitory viral infection**	**In favor of persistent viral infection**
Cytopathic effect: SARS-CoV-2 produces extensive tissue damage and cell death that reduces the chances of tumoral transformation.	Immunosuppression: -Lymphopenia and natural killer (NK) cell reduction. -Exhausted NK and CD8^+^ cells. -Diminished response of interferon. -Induction of pro-tumor cells [myeloid-derived suppressor cell (MDSC), M2 macrophages]. -Decreased CMH-1. -Alterations in autophagy.
Cell cycle stop. Subsequent apoptosis that impedes tumoral transformation.	Hyperinflammatory and protumoral responses, oxidative stress, cytokine storm: -Severe cases of COVID. -Could induce cellular proliferation, angiogenesis, DNA damage, cytoskeletal remodeling and induction of EMT.
	Direct oncogenic impact. Downregulation of tumor-suppressing proteins [retinoblastoma protein (pRB) and p53].
	Reactivation of oncogenic viruses [human papilloma virus (HPV), Epstein-Barr virus (EBV)]

Another important factor is related to air pollution as a major factor for respiratory diseases even LC. Patients by long-term exposure to polluted air leads diseases who made them less able to fight against lung infections. This factor is common in big cities and is a reasonable cause to increase the death rate by COVID-19 [13].

### Delayed diagnosis

COVID-19 has been challenging for the diagnosis of LC. Globally, healthcare activity has slowed down to comply with public health directives. In Spain, Reyes et al. [14] conducted a retrospective study of patients with LC who were diagnosed before (January to June 2019) and during the start of the pandemic (January to June 2020) and reported that new diagnoses of LC experimented a 38% decrease [non-small cell LC (NSCLC) 43%, small-cell LC (SCLC) 19%] during the last period compared to before the pandemic. More symptomatic cases, associated with worse prognosis, were diagnosed during the pandemic.

In Canada, Kasymjanova et al. [15] showed that patient referral to LC specialists and new diagnoses were reduced by 34.7% during the pandemic. The report by the Ministry of Health in Quebec also supports this idea [16].

Jazieh et al. [17] conducted a study from 356 centers in 54 countries reporting that 88% of centers had difficulties due to COVID-19 in terms of increased demand and lack of personnel.

In South Korea, Park et al. [18] studied 169 LC patients diagnosed during the pandemic (February to June 2020) and 443 patients diagnosed before (February 2017 to June 2019). They reported an increase in stage III–IV NSCLC diagnoses (74.7% in 2020 *vs.* 57.9% in 2017, 66.7% in 2018 and 62.7% in 2019; *P* = 0.011). In contrast, the percentage of patients diagnosed in early stages decreased.

In the United Kingdom, Maringe et al. [19] analyzed the impact of diagnostic delays during the pandemic and reported that COVID-19 could have increased LC deaths by 4.8%.

In Italy a real-world study [20] evaluated diagnostics delays and reported a reduction 6.9% in newly diagnosed LC in 2020 compared with 2019, the newly diagnosed were principally with stage IV in current smokers (*P* < 0.01).

### Complications in patients with LC

LC patients have a higher risk of pulmonary complications. This is explained by a number of risk factors that include old age, cardiovascular and respiratory comorbidities, lung tissue damage induced by tobacco, immunosuppression from antineoplastic therapy and corticosteroids [3, 21–24].

The LC patients with COVID-19 in Spain: GRAVID study analyzed 447 Spanish patients and showed that cancer therapies did not increase the risk of death by COVID-19. However, they reported 78.3% of hospitalizations, 62.9% of severe cases of COVID-19, 2% admitted to intensive care units and a 32.7% mortality related to tumor stage, old age and use of corticosteroids during hospitalization [22].

Previous lung surgery, radiotherapy (RT), chemotherapy and lung diseases that cause obstruction or emphysema can predispose to severe infection. Anatomical changes in the respiratory tract and lung tissue lead to an altered intra and peritumoral microenvironment that conduces to an immune infiltration with macrophages and inflammation. The subsequent release of cytokines can contribute to the development of ARDS [25, 26]. The overexpression of ACE2 in the tumor and the normal adjacent tissue could explain why patients with LC have a potentially increased risk of suffering from severe COVID-19 [27–29]. The most frequent complications in this situation include pneumonia, further bacterial or viral infections, thromboembolism, ARDS and damage to other organs such as the heart, liver or kidneys [30–32]. Furthermore, they can also develop persistent COVID [33, 34].

A retrospective analysis by Sha et al. [35] reported that the estimated risk of severe or mortal complications was 2.31 times higher than in the general population. Another study showed that the risk of death in LC patients was 4 times higher [36]. Luo et al. [3] noticed more prolonged cases of COVID-19 and more severe complications. These data are supported by the COVID-19 in patients with thoracic malignancies (TERAVOLT) study, that reported a death rate of 33%, with pneumonia and pneumonitis (79.6%), ARDS (26.8%), multiple organ disfunction syndrome (7.6%) and sepsis (5%) as the most frequent complications [37]. Given that the criteria for admission to the intensive care unit vary between centers, admission rates are variable (2–27%) and the rates of mechanical ventilation have been relatively low (7–11%) [3, 22, 37, 38].

### Radiological challenges

**Figure 1:**
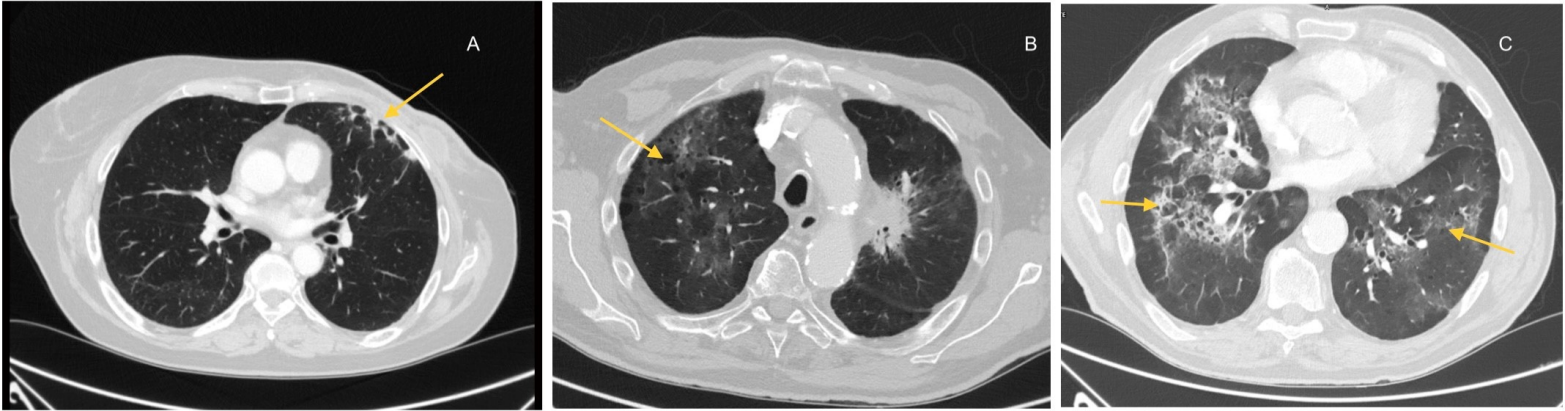
Different patterns of pneumonitis. Pneumonitis induced by (A) RT; (B) immunotherapy and (C) COVID-19

Computerized tomography (CT) scan is the imaging technique of choice to evaluate pneumonitis induced by COVID-19. Several radiological patterns have been described: from ground-glass opacities to consolidations, interlobular septal thickening, and traction bronchiectasis. These findings are usually multifocal and may involve multiple lobes [31]. In some cases, these images can be confused with other patterns induced by cancer therapies: immune checkpoint inhibitors (ICIs) and tyrosine kinase inhibitors (TKIs), detected in 4% of cancer patients (Figure 1) [31].

Given the great variety of radiological findings caused by COVID-19, the COVID-19 Reporting and Data System (CO-RADS) scale has been developed by the Dutch Radiological Society. This scale determines the probability of COVID-19 based on a series of characteristics in a thoracic CT scan, going from very low (CO-RADS 1) to very high (CO-RADS 5). There are also two additional categories: insufficient data (CO-RADS 0) and confirmed infection based on reverse transcription polymerase chain reaction (RT-PCR; CO-RADS 6; Table 2) [39]. This scale allows for a quick diagnostic orientation and for the standardization of results for an adequate diagnosis of COVID-19, together with other tests (RT-PCR, blood analysis).

**Table 2:** CO-RADS scale and its correlation with BSTI and RSNA for the diagnosis of COVID-19

CO-RADS	BSTI	RSNA
CO-RADS 0: non-interpretable	Non-interpretable	Non-interpretable
CO-RADS 1: normal/no infection	Negative for pneumonia	No COVID
CO-RADS 2: pattern consistent with other infections	Atypical pattern. Not reported for COVID-19	-
CO-RADS 3: compatible with COVID-19 but present in other diseases	Indeterminate. Not COVID-19 specific	Indeterminate
CO-RADS 4: suspicious of COVID-19	-	Probable COVID
CO-RADS 5: typical pattern of COVID-19	Typical characteristics	Classic COVID
CO-RADS 6: confirmed COVID-19 diagnosis with RT-PCR	-	-

BSTI: the BSTI statement guideline; RSNA: the RSNA expert consensus statement category; -: no correspondence in this scale

It must be noted that interobserver variability has been reported, with a Fleiss kappa of 0.47 [95% confidence interval (CI), 0.45–0.49] [39]. In case of CO-RADS 3 and with no additional conclusive tests, the authors recommended to correlate these findings with epidemiologic data, previous studies, and clinical presentation [39].

The meta-analysis by Liu et al. [40] describes high sensitivity of CO-RADS ≥ 3 (0.89; 95% CI, 0.85–0.93) and high specificity of CO-RADS ≥ 4 (0.84; 95% CI, 0.78–0.88) for the diagnosis of COVID-19.

Other scales have been developed to identify COVID-19 in CT scan, such as BSTI and the RSNA, although these are less used compared to CO-RADS scale (Table 2) [41].

For patients with emphysema and fibrosis, these scales are less specific. Therefore, it is necessary to compare with previous CT scans as well as a positive RT-PCR to stablish a correct diagnosis [24].

It is advisable to provide radiologists with information regarding the type of antineoplastic treatment that the patient is receiving. Some patterns are characteristic of certain therapies and not COVID-19, such as honeycomb lung or small centrilobular lung nodules with TKIs [24].

The screening and follow-up of lung nodules also needed to be adapted to avoid continuous visits to hospitals, as evidenced by the CHEST Expert Panel Report [7, 42].

## Persistent infection by SARS-CoV-2 and cancer risk

Non-retroviral RNA viruses usually cause an acute infection characterized by a fast viral replication and excretion, followed by a recovery phase in which the individual eliminates the virus and develops immunity against reinfection during a variable period of time. To survive, most RNA viruses have developed strategies to avoid eradication through specific mechanisms that allow for the persistent infection in some individuals, such as hepatitis C or Borna disease [43].

SARS-CoV-2 seems to have tropism for the endothelium [44], which can facilitate its persistence in tissues that are not responsible for the main symptoms of COVID-19. There have even been cases of central nervous system involvement, with positive RT-PCR in the cerebrospinal fluid [45], which suggest that persistent infection is possible in territories where immune evasion is easier. Persistent COVID-19 can be the result of low concentrations of the virus that remains trapped in certain tissues, which makes it impossible to identify through conventional tests [46]. Some studies suggest that the virus can persist in both mild and severe cases, but especially in immunosuppressed patients [47, 48]. Cases of persistent COVID-19 symptoms with RNA detection of SARS-CoV-2 and no seroconversion have also been reported [49].

Certain studies propose that long-term effects of SARS-CoV-2 infection can have oncogenic potential in terms of oncogenesis and/or tumor progression [50–54].

Generally, a virus can directly unleash cellular transformation through an external oncogene, by overactivating human oncogenes and/or by inhibiting tumor-suppressing genes such as EBV or HPV [55].

The immunosuppression, hyperinflammation and oxidative stress mediated by SARS-CoV-2 could directly or indirectly promote the development of some types of cancer in predisposed patients. Moreover, the downregulation of tumor-suppressing proteins and the possible reactivation of latent oncogenic viruses could also play a part after the infection (Table 1) [54].

On the other hand, some studies refute this association. The transitory nature of the COVID-19 infection would complicate carcinogenesis. Furthermore, the stop in the cell cycle induced by the virus and its cytopathic effect goes against the possibility of tumoral transformation. This association could be better explained by an indirect effect related to extensive and irreversible lung fibrosis that characterizes severe pneumonia from COVID-19 [56]. Further long-term studies are needed to determine the true association between SARS-CoV-2 and cancer.

## Adapted treatment during the pandemic

The COVID-19 pandemic has caused problems for screening, diagnosis and treatment of LC [57]. Different points during the pandemic have required a modification in conventional therapies for LC. In patients with pending or active treatment with COVID-19 infection, the suspension of antineoplastic therapy has been assessed in a multidisciplinary setting and in agreement with the patient, considering the risks and benefits in each specific case [57].

Surgery departments have been particularly vulnerable during the pandemic due to exposure to SARS-CoV-2 during surgical procedures, need for surgical resources for other reasons (mechanical ventilation, anesthesiologists, intensive care unit beds). In this context, many patients with resectable stage I–II LC were offered stereotactic body RT (SBRT) in 3–5 fractions as an alternative [58], with great results in terms of toxicity and local control.

In locally advanced NSCLC, treatment with concomitant radiochemotherapy (CRT) has been limited by toxicity due to side effects such as lymphopenia that could increase the risk of severe COVID-19 [59].

To adapt to the pandemic, hypofractionated thoracic RT (HTR) has been a reasonable option [60], with regimes of 15–20 fractions of 2.75 Gy to 4 Gy compared to the traditional 30–33 fractions of 2 Gy. This approach has a biologically equivalent dose and is safe and effective [61, 62]. However, the evidence for concomitant HRT and chemotherapy remains limited, which has favored sequential treatments [58, 63].

As for limited-stage (LS)-SCLC, traditional regimes are still recommended: 45 Gy in 30 fractions (2 daily fractions) [64] or normofractionation with 60–66 Gy in 30–33 fractions [65]. Although there is a lack of evidence, given the results in NSCLC, hypofractionation in SCLC has been a possibility for selected patients [66]. Recent studies suggest that starting RT with the third chemotherapy cycle has comparable efficacy, which has allowed for the adaptation of concomitant treatment when needed [67, 68].

In early-stage SCLC (T1-T2 N0M0), SBRT with doses of 50–60 Gy in 5 fractions is a good alternative to surgery [69]. Prophylactic cranial irradiation (PCI) is recommended in SCLC, but a close follow-up with brain magnetic resonance imaging was an option during the pandemic [70].

No associations have been found between the severity of COVID-19 and treatment with immunotherapy, targeted therapy or surgery. In contrast, RT and chemotherapy have reported contradictory results in different studies: some have reported an increase in mortality with CT chemotherapy [71, 72], whereas other have not [73, 74]. For RT, various studies have shown no association, but other such as Chavez-MacGregor et al. [71] have described worse prognosis related to lymphopenia [73].

Immunotherapy with ICIs have also required an adaptation. Durvalumab, a programmed death-ligand 1 (PD-L1) inhibitor used as a consolidative treatment after CRT in stage III NSCLC (restricted to patients with PD-L1 expression ≥ 1% in Europe), is usually administered every two weeks (10 mg/kg). This regimen has been occasionally modified to 20 mg/kg every four weeks during the pandemic. Furthermore, ICIs for metastatic patients have also been adapted to minimize patient contact with the hospital by choosing dosing that allow for a more spaced administration [75].

Cantini et al. [76] evaluated the safety of extended interval dosing (EID) ICIs (pembrolizumab 400 mg every 6 weeks (Q6W) or nivolumab 480 mg Q4W) and concluded ED did not increase the incidence of immune-related adverse events (irAEs) and represents a safe option also outside clinical trials. These results were confirmed in the Hijmering-Kappelle et al. [77] study, and the review of Sehgal et al. [78] confirmed that this practice will continue to be used in routine practice even long after the pandemic, particularly for patients with durable disease control. Sometimes, some chemotherapeutic agents were also given more spaced to minimize patient contact with the hospital. All these treatment adaptations are described in Table 3.

**Table 3:** Treatment changes in LC due to the COVID-19 pandemic

**NSCLC T1-T2 N0M0**	**Locally advanced NSCLC**	**Metastatic NSCLC**	**LS-SCLC**	**ES-SCLC**
More use of 3D/IMRT RT or SBRT as an alternative to surgery	Less use of surgery	Shorter and more spaced chemotherapy and ICI regimens	More use of sequential CRT	Shorter and more spaced chemotherapy and ICI regimens
	More use of sequential CRT	Single-dose palliative RT	Hypofractionated RT	Single-dose palliative RT
	Hypofractionated RT	Less use of local treatments in oligometastatic patients	PCI *vs.* close follow-up	PCI *vs.* close follow-up
	Consolidative durvalumab Q4W			

ES: extensive stage; IMRT: intensity-modulated RT

## Vaccines and seroprevalence

The European Society of Medical Oncology (ESMO) and the Spanish Society of Medical Oncology (SEOM), as well as other oncological societies recommend the vaccination of all cancer patients, especially those under active treatment [79]. This vaccination must be administered as soon as possible, ideally before the start of treatment, but it can coincide with chemotherapy as long as there is no lymphopenia or neutropenia.

Vaccination of patients with anti programmed cell death protein 1 (anti-PD-1)/PD-L1 therapy has been widely debated, but there are studies that have shown that cancer treatment can be maintained if there is a window period of more than 16 days between the first dose of the vaccine and the first dose of the drug, with no impact in effectiveness or side effects [80].

The first two vaccines approved by the European Medicines Agency (EMA) and the Food and Drug Administration (FDA) are based on messenger RNA (mRNA) BNT162b2 (Cominarty Pfizer/BioN Tech) and mRNA-1273 (Moderna) and are apt for the use in cancer patients.

The vaccine by AstraZeneca, constituted by a non-replicative viral vector (adenovirus) can be administered in immunocompromised patients.

Vaccines with an inactivated virus, developed in Russia and China can be used in cancer patients, but are not currently approved in Spain [79]. European vaccines target the S protein of SARS-CoV-2 to generate an immune response mediated by antibodies and T lymphocytes [81].

There is controversy on whether patients with cancer can generate immunity against the virus as effectively as non-cancer patients. Some studies have analyzed the antibody concentration in patients with hematological diseases and/or chemotherapy and have observed a decrease in this population [82].

However, the Massachusetts study, despite a low number of patients, concludes that the immune response is very heterogenous in patients with thoracic tumors that receive RT, and is mostly related to age and comorbidities [82]. Moreover, the study heterogeneous immunogenicity of SARS-CoV-2 vaccines in cancer patients receiving RT (SOLID) is a prospective longitudinal multicenter study in Spain that includes 1,500 patients. The objective was to determine the seroprevalence in patients with LC. They reported that immunity against SARS-CoV-2 does not seem to be altered in LC and can persist for more than four months as in the general population. No increase in mortality was observed in this group [83].

Although there is no definitive evidence yet and larger studies are required, vaccination and booster doses are recommended to increase antibody concentration against SARS-CoV-2 [79, 83, 84].

## Conclusions

The COVID-19 pandemic is still ongoing and cases around the world continue to rise. Patients with LC, due to their vulnerability, have been affected in terms of delayed diagnosis, increased hospitalizations, and modification/suspension of antineoplastic therapies. However, a better understanding of COVID-19 diagnosis (CT scan scales, RT-PCR), the good results of adapted cancer treatments, vaccination, and improved knowledge about the physiopathology of the infection in cancer patients are modifying the morbidity and mortality of these patients towards more favorable results. Undoubtedly, the COVID-19 pandemic has generated a rebuild, the oncological activities and the recommendations to prioritize anti-tumor treatments and combined with a COVID-19 pandemic and LC is not the exception.

## Abbreviations

ACE2: angiotensin-converting enzyme 2
ARDS: acute respiratory distress syndrome
CI: confidence interval
COVID-19: coronavirus disease 2019
CRT: radiochemotherapy
CT: computerized tomography
ICIs: immune checkpoint inhibitors
LC: lung cancer
LS: limited-stage
NSCLC: non-small cell lung cancer
PCI: prophylactic cranial irradiation
PD-L1: programmed death-ligand 1
RT: radiotherapy
SARS-CoV-2: severe acute respiratory syndrome coronavirus 2
SBRT: stereotactic body radiotherapy
SCLC: small-cell lung cancer

## Declarations

### Copyright

© The Author(s) 2023.
